# GPR156 is required in sensory hair cells for proper auditory and vestibular function

**DOI:** 10.1038/s41598-025-34476-4

**Published:** 2026-01-17

**Authors:** Amandine Jarysta, Brandie Morris Verdone, Elli I. Hartig, Kathleen E. Cullen, Basile Tarchini

**Affiliations:** 1 The Jackson Laboratory, Bar Harbor, ME 04609 USA; 2 Department. of Biomedical Engineering, Johns Hopkins University, Baltimore, MD 21205 USA; 3 Department of Otolaryngology-Head and Neck Surgery, Johns Hopkins University, Baltimore, MD 21205 USA; 4 Department of Neuroscience, Johns Hopkins University, Baltimore, MD 21205 USA; 5 Kavli Neuroscience Discovery Institute, Johns Hopkins University, Baltimore, MD 21205 USA; 6 Tufts University School of Medicine, Boston, MA 02111 USA

**Keywords:** Cell biology, Developmental biology, Genetics, Neuroscience

## Abstract

Proper orientation of the apical cytoskeleton in auditory and vestibular hair cells is essential for their sensory function. A recently identified regulator of hair cell orientation is the G protein-coupled receptor GPR156, which signals through inhibitory heterotrimeric G proteins. In hair cells expressing the transcription factor EMX2, GPR156 is apically enriched and polarized at cell junctions. There, GPR156 signaling reverses the interpretation of tissue-level core planar cell polarity cues, effectively reversing the orientation of *Emx2*-positive compared to *Emx2*-negative hair cells. This mechanism establishes key anatomical features, such as the correct alignment of auditory outer hair cells and the line of polarity reversal in the otolith organs of the vestibular system. Null mice with constitutive *Gpr156* inactivation exhibit severe hearing loss, mirroring congenital hearing impairment in human patients with homozygous *GPR156* variants. These null mutants also display impaired swimming and vestibulo-ocular reflexes, although the nature of these vestibular deficits differs from those reported in *Emx2* mutants. Here, to determine the extent to which functional deficits arise from hair cell misorientation, we conditionally inactivated *Gpr156* in postmitotic hair cells in the inner ear. This targeted deletion approach recapitulated the misorientation phenotype observed in null mutants. Notably, 30–40% of cochlear and utricular hair cells affected in the null background retained normal orientation in conditional mutants, likely due to the later timing of *Gpr156* inactivation. Despite reduced efficiency, conditional mutants exhibited similar, albeit predictably milder, auditory and vestibular dysfunction. As hair cells can carry out mechano-electrical transduction without GPR156, we conclude that sensory deficits mainly result from its essential role in hair cell orientation.

## Introduction

GPR156 is an orphan class C GPCR most closely related to GABA metabotropic receptors. Early studies ruled out the possibility that GPR156 is an alternative GABA receptor, leaving its physiological function unknown^1,2^. A first functional role was later identified in the inner ear, where GPR156 is required for the correct orientation of a subset of hair cells^3^. Hair cells are directional sensors for sound in the cochlea and for head movement in the vestibular organs. Each hair cell develops a highly asymmetric apical cytoskeleton that includes the hair bundle, a graded array of mechanosensitive, actin-based protrusions^4^. While each hair bundle is inherently directional^5^, the accuracy of mechanosensory responses also depends on the precise orientation adopted by hair cells during development^6^.

**Fig. 1:**
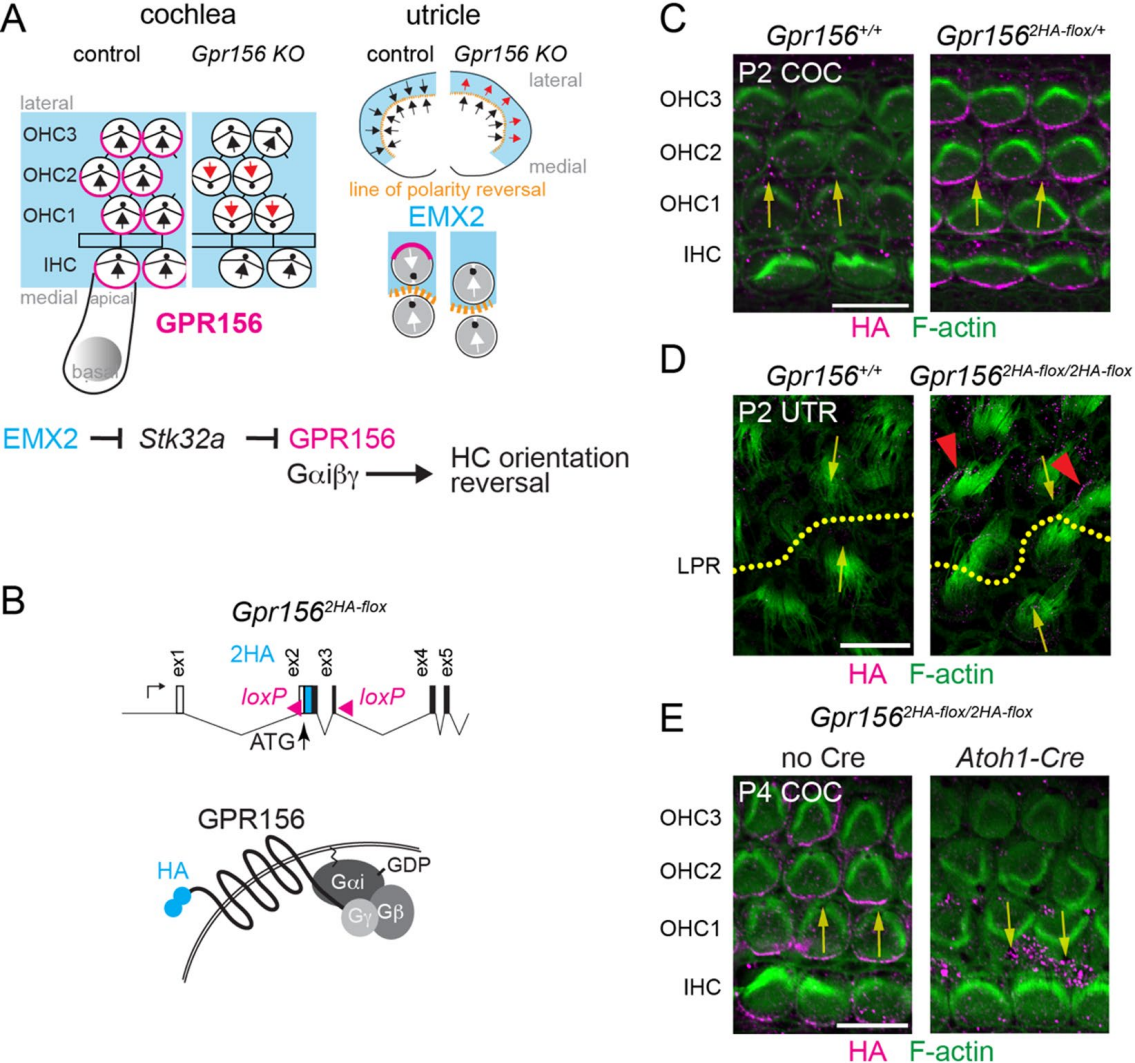
Dual reporter and conditional inactivation *Gpr156* mouse model. **(A)** Schematic of GPR156 enrichment (magenta), *Emx2* expression (blue) and constitutive *Gpr156* knock-out phenotypes in the cochlea (left) and utricle (right) based on previous studies. **(B)** New mouse allele where the first *Gpr156* coding exons (ex 2–3) are flanked by *lox*P sites and two HA tags are inserted in frame N-terminal. **C-D)** P2 cochlea (C) and utricle (D) immunolabeled for HA along with F-actin (phalloidin conjugate). HA signal recapitulates known GPR156 polarized enrichment in *Gpr156*^*2HA − flox*^, but not in wild-type littermate samples. Specifically, a medial junctional distribution in all cochlear hair cells (C) and a lateral junctional distribution in utricular hair cells in the lateral extrastriolar region above the line of polarity reversal (D, red arrowheads). **E)** P4 cochleae labeled for HA and F-actin. Note how OHC1-2 are only aberrantly inverted in orientation upon Cre recombination in *Gpr156*^*2HA − flox*^ homozygotes. Yellow arrows indicate hair cell orientation. COC, cochlea; UTR, utricle; LPR, line of polarity reversal. Scale bars: 10 μm.

In the otolith vestibular organs that detect linear acceleration and gravity, macular hair cells are organized into two populations with opposite orientations across a virtual line of polarity reversal (Fig. 1A, right). This anatomical divide is established by the selective expression of the transcription factor EMX2 in one population only^7,8^. EMX2 represses transcription of the kinase *Stk32a*, which normally prevents apical enrichment and planar polarization of GPR156 at the apical junction^9^. As a result, in *Emx2*-positive (*Emx2+)* hair cells, apical GPR156 can signal through inhibitory G proteins^10^, reversing how these cells interpret asymmetric core planar cell polarity cues at apical cell-cell junctions during symmetry breaking. In constitutive *Gpr156* mutants, the *Emx2* + population fails to reverse and instead adopts the same range of orientations as the *Emx2-*negative (*Emx2-)* population, effectively abrogating the line of polarity reversal. These patterning defects are accompanied by functional impairments in swimming and vestibulo-ocular reflexes that specifically engage the otolith organs^11,12^.

The auditory epithelium is entirely derived from the *Emx2* lineage^13^, and accordingly, GPR156 is expressed and planar-polarized in all auditory hair cells (Fig. 1A, left). However, in constitutive *Gpr156* mutants, only outer hair cells (OHC) in rows 1 and 2 adopt an inverted orientation, while inner hair cells (IHCs) and OHCs in row 3 are less affected^3^. This correlates with severe hearing deficits, specifically markedly elevated auditory brainstem response (ABR) thresholds at 8, 16 and 32 kHz. In addition, thresholds for distortion product otoacoustic emissions (DPOAEs) are also elevated, indicating impaired OHC function^3^. These phenotypes illuminate the etiology of congenital hearing loss reported in multiple families with homozygous *GPR156* variants^14–16^.

Constitutive *Gpr156* and *Emx2* mutants exhibit similar anatomical defects in otolith organs, consistent with their epistatic relationship^3,7^. *Gpr156* expression is limited to hair cells within the sensory epithelium whereas *Emx2* is more broadly expressed, including in supporting cells^3,17^. Nevertheless, conditional inactivation of *Emx2* in hair cells using the *Gfi1-Cre* driver recapitulates failed reversal in orientation observed in the constitutive null *Emx2* allele^7^. In contrast to *Emx2* null mutants, which do not survive past birth and thus preclude behavioral testing, conditional *Emx2* mutants survive and show mild vestibular deficits that appear somewhat distinct from those observed in *Gpr156* mutants^17^. Notably, in swimming tests, *Emx2* mutants exhibit a frantic behavior distinct from the more severe tumbling and drowning phenotypes observed in *Gpr156* null mutants. This behavioral distinction may suggest that GPR156 has additional roles outside the inner ear sensory epithelia that further impact vestibular function. This possibility has been difficult to resolve due to limitations in comparative testing. For instance, vestibulo-ocular reflexes were assessed in *Gpr156* null but not in *Emx2* mutants^11^, whereas vestibular sensory evoked potentials (VsEPs) were evaluated in *Emx2* but not *Gpr156* mutants^17^. Additional confounding factors also exist. Orientation-selective afferent innervation is disrupted in otolith organs when *Emx2* is inactivated in hair cells, whereas constitutive *Gpr156* inactivation does not impact afferent patterning^11,17^.

We developed a conditional mouse model to inactivate *Gpr156* in a cell- and time-specific manner. Using this new resource, our objective was to limit inactivation to post-mitotic hair cells and provide proof-of-concept evidence that the anatomical and functional defects observed in the constitutive null are recapitulated. Since this is indeed the case, and given that mechano-electrical transduction in hair cells does not depend on GPR156^3,11^, our findings support the conclusion that deafness and vestibular deficits in *Gpr156* mutants primarily stem from hair cell misorientation.

## Results

### Dual reporter / conditional knock-out *Gpr156* mouse model

Using CRIPSR/Cas9 and a plasmid-based donor vector, we generated a new mouse strain where two HA tags were inserted in phase N-terminal to the *Gpr156* coding sequenced in exon 2. The resulting protein should present two HA epitopes on the extracellular side of the plasma membrane (Fig. 1B). Exons 2 and 3 were also flanked by *loxP* sites, allowing Cre-based recombination to both remove HA signals and inactivate *Gpr156.* We named this allele *Gpr156*^*2HA − flox*^. We harvested *Gpr156*^*2HA − flox*/+^ neonatal cochleae and immunolabeled the auditory epithelium with an HA antibody. As expected, HA signals formed a medial crescent at the apical hair cell junction (Fig. 1C), as observed with GPR156 antibodies^3^. This pattern was not observed in wild-type littermates (Fig. 1C). We also verified that HA immunolabeling recapitulated GPR156 distribution in utricular hair cells in the lateral extrastriolar region (Fig. 1D). Next, to verify that *Gpr156* loss-of-function is achieved upon Cre recombination, we labeled F-actin and HA at the hair cell surface in homozygous cochleae (*Gpr156*^*2HA − flox/2HA−flox*^). As expected, OHC1-2 misorientation characteristic of the *Gpr156* null allele^3^ and loss of HA signals were only observed in Cre-positive, but not Cre-negative homozygotes (Fig. 1E). This verifies that the HA and *loxP* insertions do not severely disrupt gene function. In conclusion, *Gpr156*^*2HA − flox*^ is suitable to serve as a knock-in reporter to visualize or purify GPR156 with HA antibodies, as well as a conditional allele for tissue- and time-specific *Gpr156* inactivation.

### *Gpr156* inactivation in the inner ear using *Foxg1*^*Cre*^

**Fig. 2:**
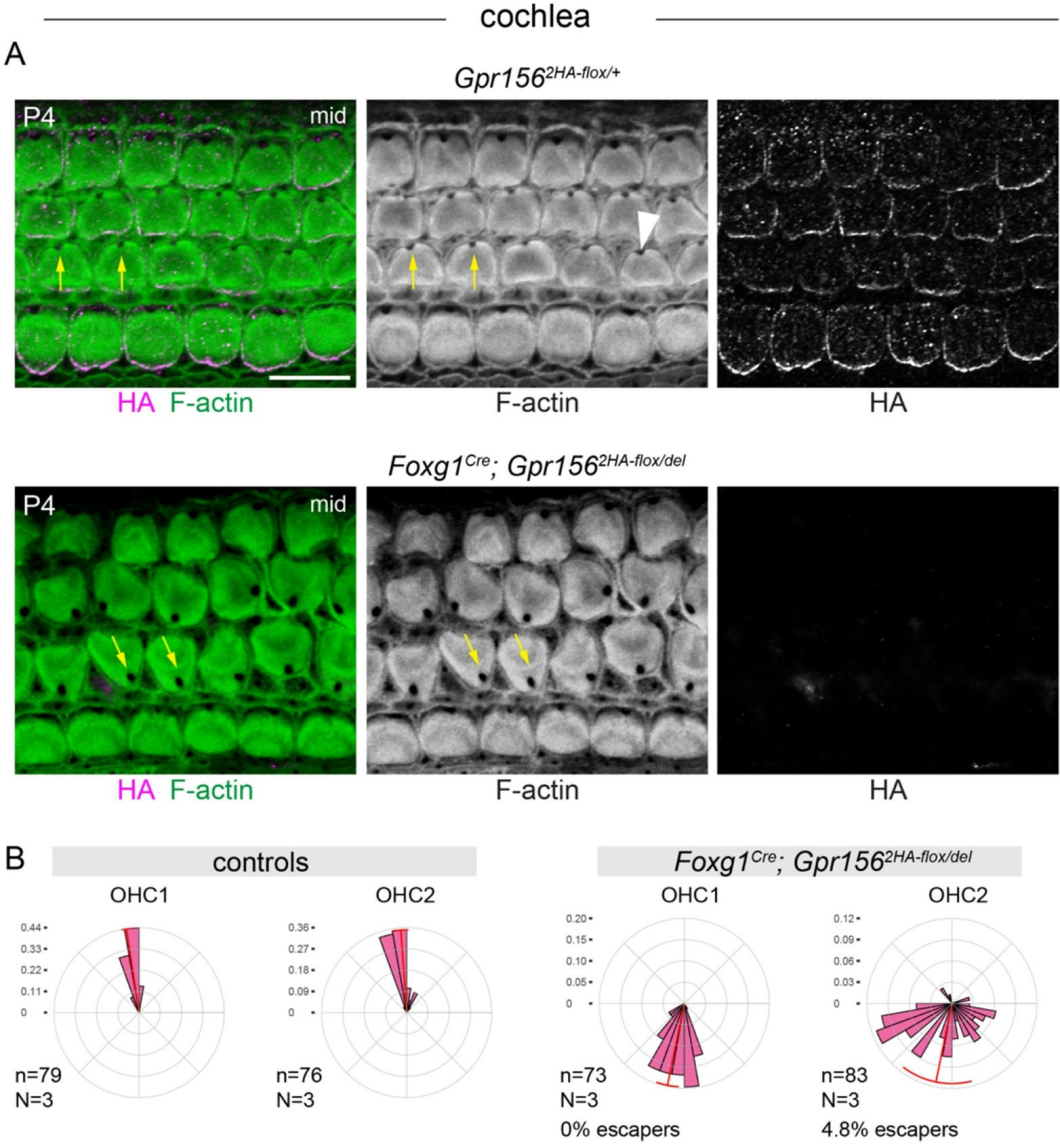
Hair cell orientation defects in conditional *Gpr156* mutant cochlea using the early *Foxg1*^*Cre*^ driver. **(A)** P4 cochleae labeled with HA and F-actin. Conditional mutants (bottom) lack HA signals and show OHC1-2 misorientation (yellow arrows). The fonticulus is indicated by an arrowhead. **(B)** Circular diagram of OHC1-2 orientation as a frequency distribution. n and N respectively indicate the number of hair cells and animals analyzed. While control OHC1-2 point laterally, conditional mutant OHC1-2 are inverted and point medially. Escapers are defined as hair cells pointing laterally (0-180° where 0° is towards the cochlear base and 90° is lateral). In circular graphs, bin size is 10 degrees and the red arc position and span respectively indicate the circular mean and circular standard deviation of the angle distribution. Scale bars: 10 μm.

Next, we crossed the *Gpr156*^*2HA − flox*^ allele and the null allele (*Gpr156*^*del*^ ; see Methods for details)^3^ to increase gene deletion efficiency by limiting recombination to one parental locus (*Gpr156*^*2HA − flox/del*^). We bred in the well-characterized *Foxg1*^*Cre*^ driver expressing Cre in the otic vesicle for early inactivation in the developing inner ear^18^. In the cochlea, *Foxg1*^*Cre*^; *Gpr156*^*2HA − flox/del*^ conditional mutants mimicked the null allele, showing inverted OHC1 and OHC2 based on the V-shape of the hair bundle and the position of the fonticulus, the region devoid of F-actin around the basal body (Fig. 2A)^3^. As expected, all mutant hair cells also lacked the HA medial crescent observed in littermate controls. Quantification of OHC1-2 orientation confirmed consistent inversion compared to control littermates, with a very low proportion of “escapers” defined as hair cells adopting a generally lateral orientation (OHC1: 0% escapers; OHC2: 4.8% escapers; lateral: 0-180° in a reference system where 0° is towards the cochlear base and 90° is lateral). Of note, constitutive *Gpr156* mutants also show OHC2 escapers^3^.

**Fig. 3:**
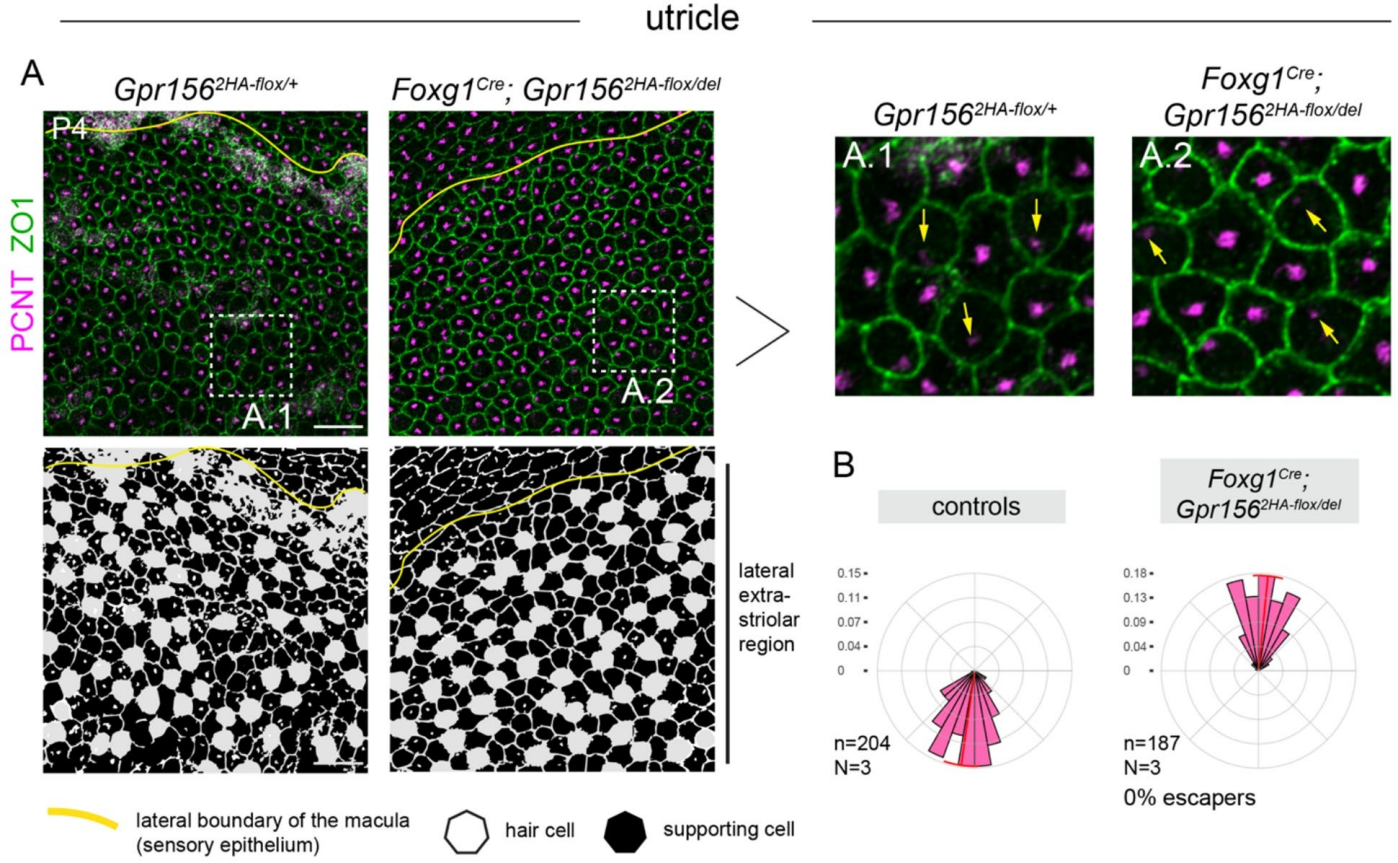
Hair cell orientation defects in conditional *Gpr156* mutant utricle using the early *Foxg1*^*Cre*^ driver. **(A)** P4 utricles labeled for pericentrin (PCNT) to reveal the basal body and ZO1 to reveal apical junctions. Conditional mutants show inverted hair cells that point laterally (up) throughout the lateral extrastriolar region compared to control hair cells that point medially (down) based on the position of the basal body. Black and white panels (bottom) indicate the position of hair cells (white) and supporting cells (black) based on F-actin and βII-spectrin labels (not shown). Insets are magnified to the right (A.1, A.2). Yellow arrows indicate orientation for select hair cells. **(B)** Circular diagrams of hair cell orientation in the lateral extrastriolar region. All hair cells in the mutant are inverted and generally point laterally. n and N respectively indicate the number of hair cells and animals analyzed. Escapers are defined as hair cells pointing medially (180–360° where 90° is lateral). Bin size is 10 degrees and the red arc position and span respectively indicate the circular mean and circular standard deviation of the angle distribution. Scale bars: 10 μm.

In the utricle, hair cells expressing *Emx2* in the lateral extrastriolar region usually point medially towards the line of polarity reversal based on the off-center position of the basal body labeled by pericentrin (PCNT) (Fig. 3A). In *Foxg1*^*Cre*^ conditional mutants, lateral extrastriolar hair cells were consistently inverted, pointing laterally (Fig. 3B; 0% escapers). These results show that early *Gpr156* inactivation in the otic vesicle recapitulates hair cell misorientation reported in the constitutive null allele. In both cases, GPR156 is absent when hair cells break central symmetry, and consequently, the normal reversal in orientation characteristic of *Emx2*-positive hair cells does not occur^3^.

### *Gpr156* inactivation in cochlear and vestibular hair cells using *Atoh1-Cre*

**Fig. 4:**
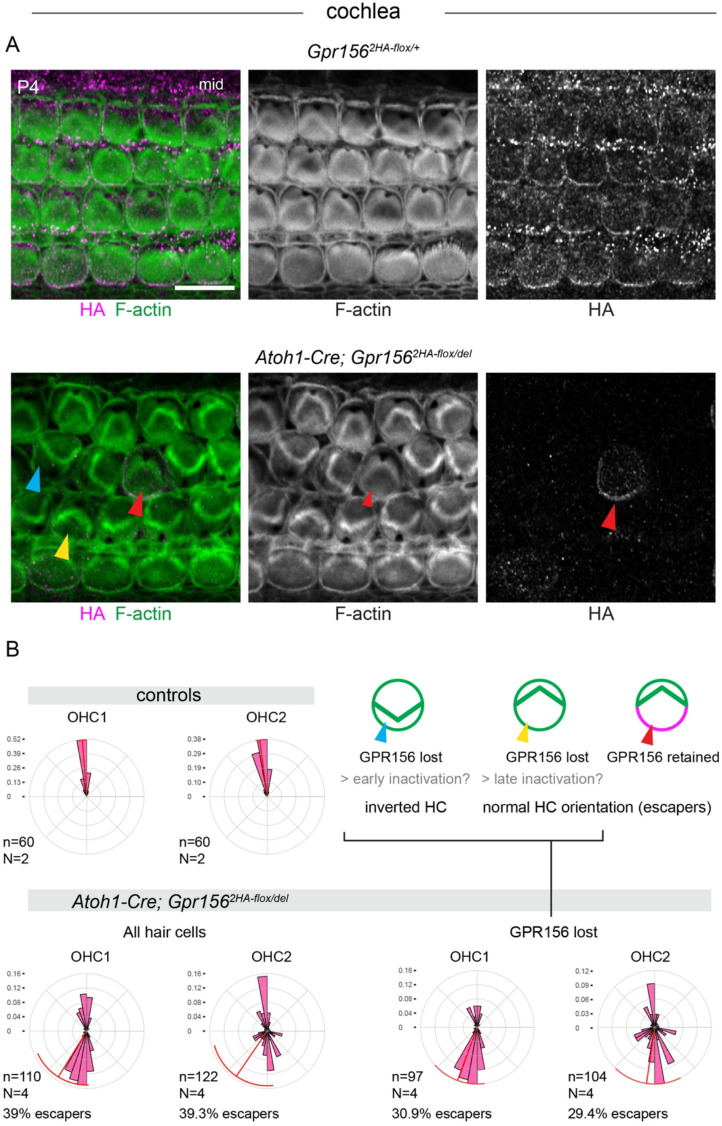
Hair cell orientation defects in conditional *Gpr156* mutant cochlea using the late *Atoh1-Cre* driver. **(A)** P4 cochleae labeled with HA and F-actin. Note how OHC1-2 in conditional mutants (bottom) can be misoriented and lack HA signals (cyan arrowhead), or be “escapers” with a normal orientation and either missing (yellow arrowhead) or retained 2HA-GPR156 (red arrowhead; schematized in B). **(B)** Circular diagram of OHC1-2 orientation as a frequency distribution. n and N respectively indicate the number of hair cells and animals analyzed. While control OHC1-2 point laterally, a large proportion of conditional mutant OHC1-2 are inverted and point medially. Note how excluding hair cells where *Gpr156* is retained (HA-positive, red arrowheads) limits the proportion of escapers defined as OHC1-2 with a lateral orientation (0-180° where 0° is towards the cochlear base and 90° is lateral). In circular graphs, bin size is 10 degrees and the red arc position and span respectively indicate the circular mean and circular standard deviation of the angle distribution. Scale bars: 10 μm.

To limit *Gpr156* inactivation in the inner ear to hair cells, we next bred *Gpr156*^*2HA − flox/del*^ with an *Atoh1-Cre* driver^19^. *Atoh1* is first expressed in post-mitotic prosensory cells that will differentiate into hair cells, although some early expressing cells also adopt a supporting cell fate^20,21^. In contrast to early *Foxg1*^*Cre*^, later *Atoh1-Cre* inactivation resulted in more variable outcomes. Most cochlear OHC1-2 were inverted as in the null and *Foxg1*^*Cre*^ models (Fig. 4A, cyan arrowhead), but some OHC1-2 showed a normal lateral orientation. Based on HA immunolabeling, these escapers represented two distinct populations: HA-positive hair cells where the GPR156 protein was still present (Fig. 4A, red arrowhead), or HA-negative hair cells that lacked detectable GPR156 signals at postnatal day 4 (P4), the stage harvested (Fig. 4A, yellow arrowhead). Considering all OHC1-2 cells, conditional mutants showed a biphasic distribution where cells were either inverted (~ 270°) compared to control littermates, or normally oriented laterally (90°; escapers: ~39% of OHC1-2) (Fig. 4B). When we excluded HA-positive cells that retained GPR156 function, the proportion of escapers was reduced to 30.9% (OHC1) and 29.4% (OHC2). We conclude that the *Atoh1-Cre* transgene achieves a timely inactivation of GPR156 in about 61% of OHC1-2. In about 30% of OHC1-2, however, *Gpr156* inactivation probably occurs past the stage where hair cells break symmetry to adopt a lateral orientation (E17.5 at the cochlear mid-apex; see Supplementary Fig. 5 in^3^. In these cells, GPR156 is still able to reverse the orientation of the basal body migration from medial to lateral as it normally does, likely explaining why some escapers adopted a normal lateral orientation but eventually lost GPR156 a week later at P4 (Fig. 4A; yellow arrowheads). Finally, in about 9% of OHC1-2, *Atoh1-Cre* did not achieve recombination at the *Gpr156* locus by P4.

**Fig. 5:**
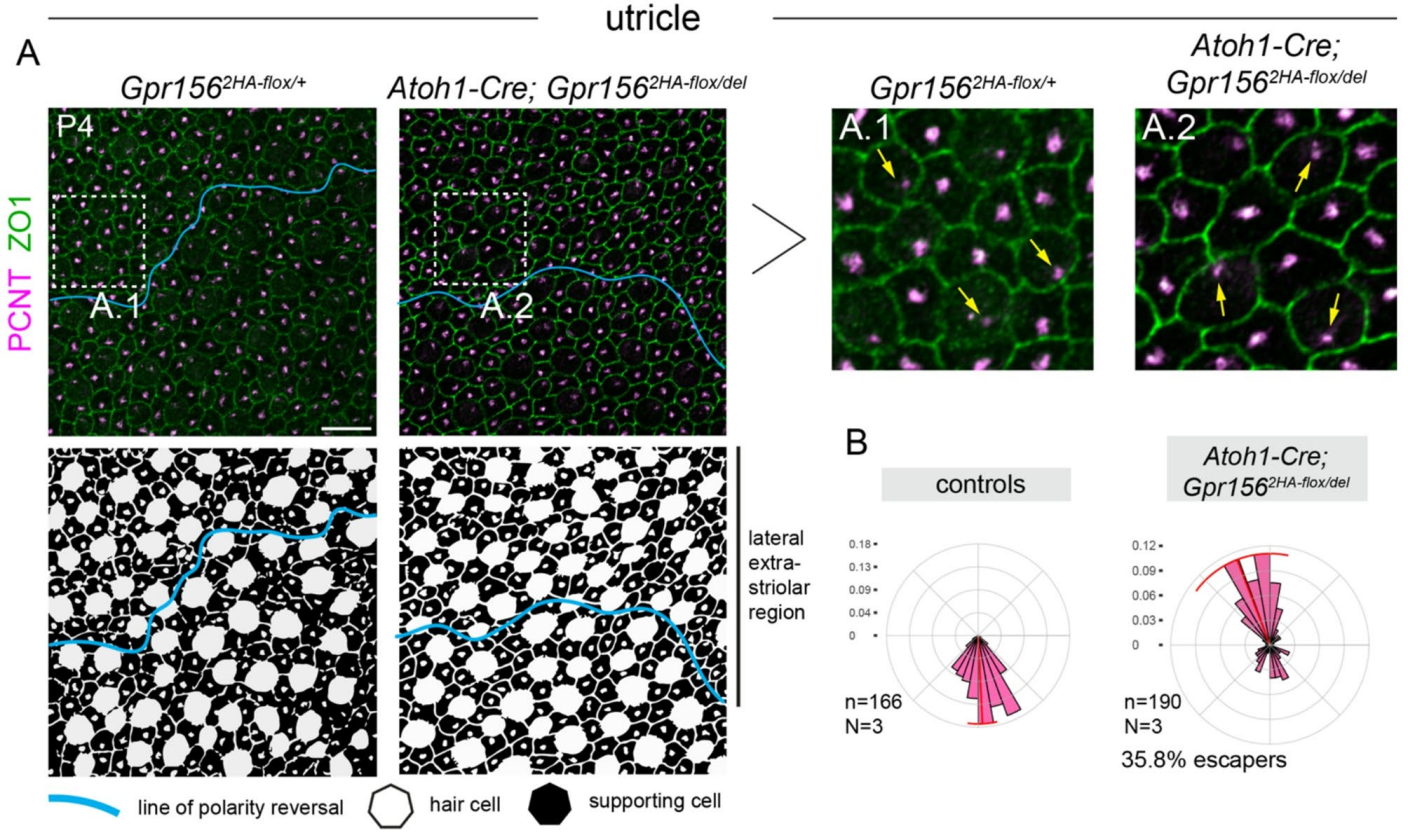
Hair cell orientation defects in conditional *Gpr156* mutant utricle using the late *Atoh1-Cre* driver. **(A)** P4 utricles labeled for pericentrin (PCNT) to reveal the basal body and ZO1 to reveal apical junctions. Conditional mutants show a mixture of inverted hair cells that point laterally (up) and non-inverted hair cells that point medially (down) throughout the lateral extrastriolar region based on the position of the basal body. Black and white panels (bottom) indicate the position of hair cells (white) and supporting cells (black) based on F-actin and βII-spectrin labels (not shown). Insets are magnified to the right (A.1, A.2). Yellow arrows indicate orientation for select hair cells. **(B)** Circular diagrams of hair cell orientation in the lateral extrastriolar region. n and N respectively indicate the number of hair cells and animals analyzed. Escapers are defined as hair cells pointing medially (180–360° where 90° is lateral). As we could not reliably distinguish HA^+^ vs. HA^−^ cells, escapers may have either lost or retained GPR156. Bin size is 10 degrees and the red arc position and span respectively indicate the circular mean and circular standard deviation of the angle distribution. Scale bars: 10 μm.

We conducted a similar analysis in the lateral extrastriolar region of the utricle in *Atoh1-Cre* conditional mutants, and essentially observed the same outcome. While hair cells in littermate controls pointed medially, most hair cells were inverted and abnormally pointed laterally in mutants (Fig. 5A-B). However, 35.8% of mutant hair cells were escapers, adopting a normal medial orientation similar to control littermates (Fig. 5A-B). In the utricle, low signal intensity (Fig. 1D) prevented us from reliably determining whether each hair cell was HA-positive or HA-negative, precluding a breakdown comparable to that shown for the cochlea (Fig. 4B). In summary, *Atoh1-Cre* allows to largely limit *Gpr156* inactivation to hair cells, with the caveat that the late loss-of-function produces a partial phenotype- a classic example of incomplete penetrance.

We reasoned that incomplete penetrance might still result in functional deficits, albeit milder in nature compared to those observed in the null allele. We thus next conducted auditory and vestibular assessments in *Atoh1-Cre; Gpr156*^*2HA − flox/del*^ conditional mutants.

### Auditory dysfunction in *Gpr156* conditional mutants using *Atoh1-Cre*

**Fig. 6:**
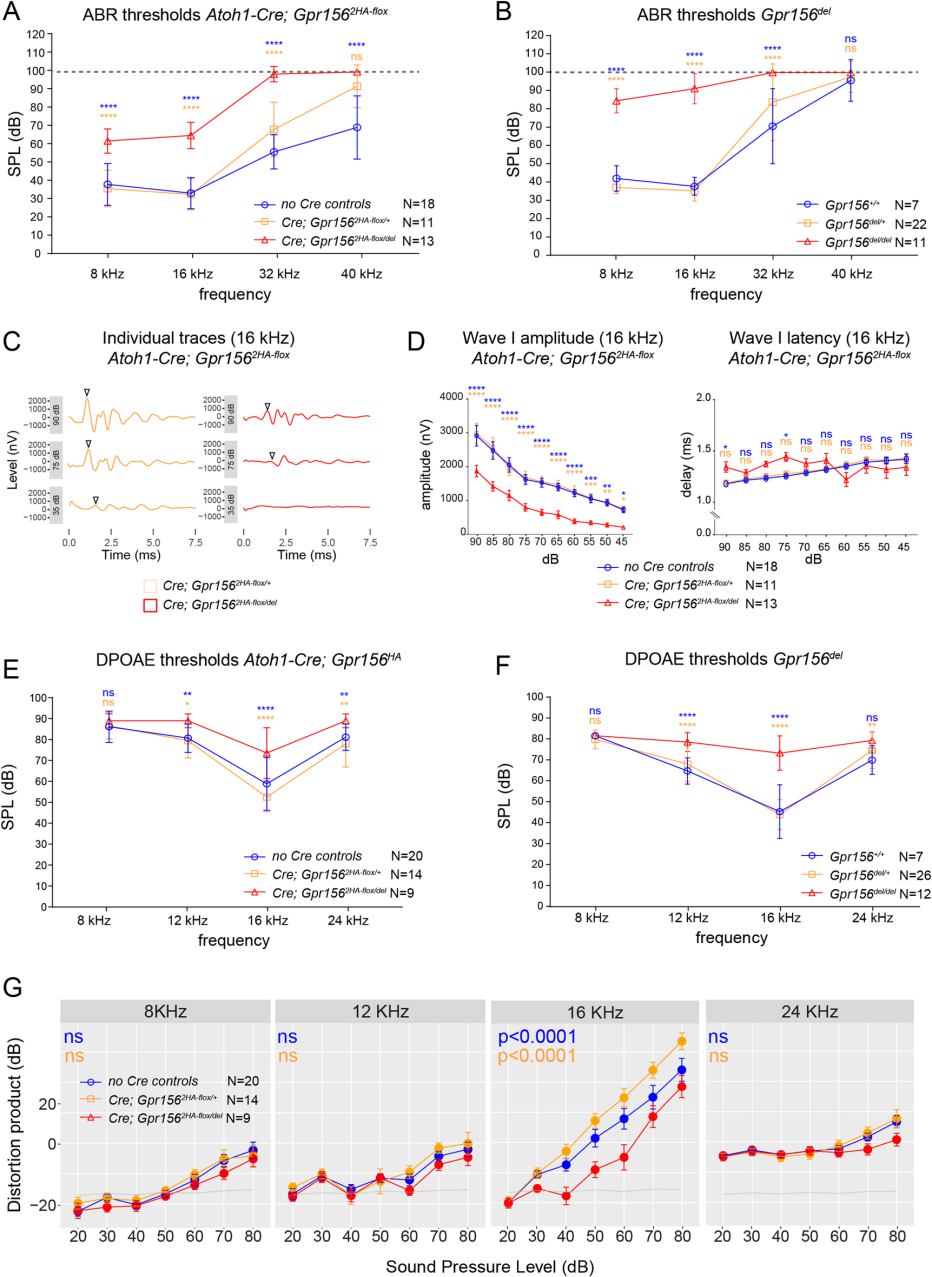
Auditory brainstem response and distortion product measurements in conditional and constitutive *Gpr156* mutants. **A-B)** ABR thresholds for the genotypes indicated in *Atoh1-Cre* conditional (A) and constitutive (B) *Gpr156* mutants. **C)** Example ABR traces at 16 kHz for one control (orange) and conditional mutant (red) for 90, 75, and 35 dB stimuli. **D)** Wave I amplitude and latency measured from individual ABR traces at 16 kHz for 45 to 90 dB stimuli in the conditional *Gpr156* mutants. **E-F)** Distortion product otoacoustic emission (DPOAE) thresholds for the genotypes indicated in *Atoh1-Cre* conditional (E) and constitutive (F) *Gpr156* mutants. **G)** DPOAE graphs for conditional *Atoh1-Cre* mutants and control littermates. Conditional mutants where *Gpr156* inactivation is limited to hair cells in the inner ear show auditory deficits similar, but less pronounced than in constitutive mutants (A-B, E-F). The constitutive mutant dataset was published in (Kindt et al., 2021) and is shown here for comparison only (B, F). N indicates the number of animals analyzed. Error bars are SD for A, B, E, F and SEM for D and G. For A and E-G, no Cre control genotypes are *Gpr156*^*2HA − flox/+*^ and *Gpr156*^*2HA − flox/del*^. Two-way ANOVA with Sidak’s multiple comparison except for G, where three-way ANOVA with Tukey’s multiple comparison was applied. Blue and orange p values indicate significance of the mutant condition (red) compared to no Cre and Cre control genotypes, respectively. **** *p* < 0.0001, *** *p* < 0.001, ** *p* < 0.01, * *p* < 0.05, ns *p* > 0.05 (not significant). SPL, sound pressure level; dB, decibel.

We recorded auditory brainstem response (ABR) in *Atoh1-Cre* conditional mutants and control littermates at ~ 8 weeks of age using pure tone stimuli at 8, 16, 32 and 40 kHz. ABR measures the neural activity evoked by sound stimuli in the auditory nerve and brainstem. Compared to controls lacking Cre expression (*Gpr156*^*2HA − flox/+*^ or *Gpr156*^*2HA − flox/del*^) or Cre controls (*Atoh1-Cre; Gpr156*^*2HA − flox/+*^), conditional mutants showed significantly elevated ABR thresholds at all frequencies tested, and complete deafness at 32 and 40 kHz (Fig. 6A). This profile is reminiscent of ABR thresholds previously published in the constitutive *Gpr156* mutants at ~ 4 weeks of age at the same frequencies, where complete deafness was also limited to higher frequencies^3^ (original data presented in Fig. 6B). At 8 and 16 kHz, thresholds were less elevated in the conditional compared to constitutive mutant, consistent with a milder auditory defect. To confirm auditory defects in conditional mutants, we measured and graphed ABR wave I amplitude and latency at 16 kHz (Fig. 6C-D). Amplitudes were significantly reduced in conditional mutants compared to littermate controls at all sound pressure levels (SPL) while latency was not significantly affected (Fig. 6D).

We reached a similar conclusion when examining more specifically OHC response by recording distortion product otoacoustic emissions (DPOAEs). DPOAEs are sound emitted by the inner ear in response to two pure tones presented simultaneously, and this response largely depends on OHC integrity. At stimulus frequencies of 12, 16 and 24 kHz, DPOAEs thresholds appeared elevated in the *Atoh1-Cre* conditional mutant (Fig. 6E), but significance was reduced compared to values reported previously in the null mutant (Fig. 6F)^3^. To confirm DPOAE defects in conditional mutants, we measured and graphed the distortion products across different sound pressure levels by stimulus frequency. The distortion products trended as lower in conditional mutants compared to controls at all frequencies tested, with significant reduction at 16 kHz (Fig. 6G). Together, these results suggest that incompletely penetrant OHC1-2 anatomical defects in the *Atoh1-Cre* conditional mutant cochlea are sufficient to produce a hearing loss profile similar, if less pronounced, to constitutive *Gpr156* mutants.

### Vestibular behavior defects in *Gpr156* conditional mutants using *Atoh1-Cre*

**Fig. 7:**
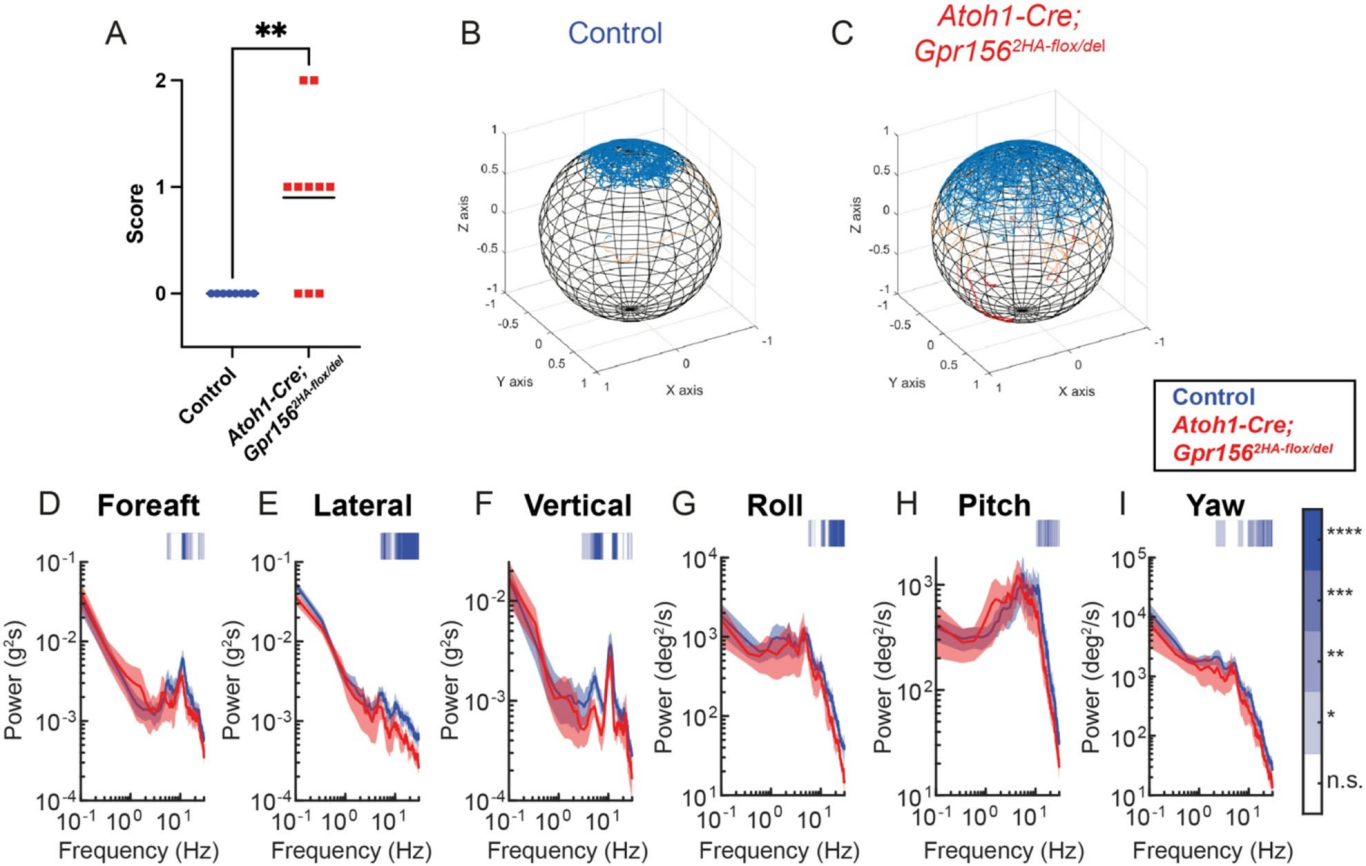
Conditional *Gpr156* mutants display hypoactivity during volitional swim. **(A)** Scored swim assessment of *Atoh1-Cre* conditional mutants compared to controls. 0 indicates completion of swim trial without intervention, 1 indicates intermittent periods of low or no activity in the animal, and 2 indicates failure to complete the trial. *N* = 8 controls, 10 mutants. *P* = 0.0039, Welch’s t test. **(B)** Spherical representation of a control animal’s head orientation during instrumented swim. Blue represents forehead vector at a 45° pitch to be nominally aligned with gravity as described in^12^. Orange regions of the trace represent a 66°-90° deviation from upright, while red represents a deviation greater than 90°. **(C)** Same as in B but for a conditional mutant animal. **D-I)** Head motion power spectra in the translational acceleration **(D-F)** and rotational velocity **(G-I)** domains during instrumented swim. *N* = 5 controls (blue), 6 mutants (red). Blue bars indicate a higher motion power in controls during that frequency window. **** *p* < 0.0001, *** *p* < 0.001, ** *p* < 0.01, * *p* < 0.05, ns *p* > 0.05 (not significant). Mean ± shaded SEM.

We next tested whether *Atoh1-Cre* conditional mutant mice displayed impairments in vestibulomotor function. Previous work showed that constitutive *Gpr156* knockout mice exhibit marked deficits in swimming and off-vertical axis rotation (OVAR), consistent with otolith-specific functional disruption^11,12^. We therefore asked whether similar deficits would be observed in *Atoh1-Cre* conditional mutants. In the scored swim test, control mice maintained upright posture and swam continuously throughout the trial without intervention. In contrast, *Atoh1-Cre* conditional mutants exhibited mild but consistent impairments (Fig. 7A), remaining upright but displaying intermittent freezing and general hypoactivity with minimal need for human rescue (Fig. 7A, red symbols). These impairments differed from those observed in constitutive *Gpr156* mutant mice, which frequently tumbled and lost postural control. We next used a validated instrumented swim test^12^ to further assess postural dynamics during swim. Notably, inertial measurement unit (IMU) recordings revealed that *Atoh1-Cre* conditional mutants maintained an upright head orientation but exhibited greater variability in their orientation over time (Fig. 7C) compared to controls (Fig. 7B). This increased variability is consistent with the intermittent floating and reduced propulsive motion observed during the swim trials. Further, power spectral analysis revealed a general reduction in movement dynamics in conditional mutants, particularly in mid-high frequency ranges in translational acceleration (Fig. 7D-F) and rotational velocity (Fig. 7G-I). Together, these results show that *Atoh1-Cre* conditional mutants exhibit subtle but measurable swimming deficits, contrasting with the severe postural instability seen in constitutive mutants^12^.

**Fig. 8:**
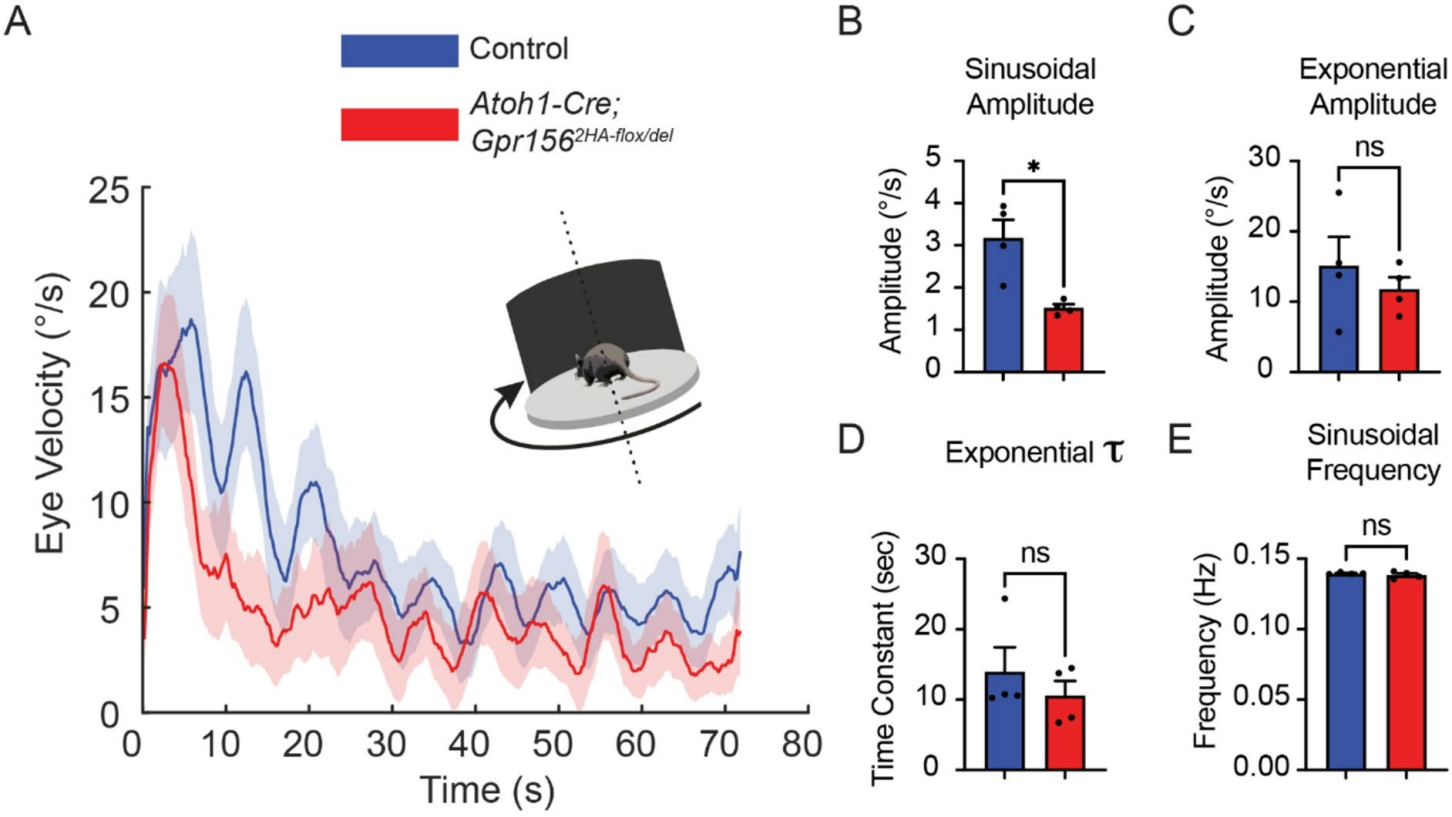
Altered otolith response during off-vertical axis rotation in conditional *Gpr156* mutants. **(A)** Average horizontal eye velocities (Mean ± shaded SEM) during a 72-second long off-vertical axis rotation for *Atoh1-Cre* conditional mutants and control mice. Inset shows schematic of the OVAR stimulus. **(B)** Sinusoidal amplitude of the slow phase eye velocity, showing a significant reduction in mutants (*p* = 0.02). **(C)** Exponential amplitude of the initial velocity decay, showing no significant difference between groups (*p* = 0.69). **(D)** Exponential time constant (τ) did not differ between groups (*p* = 0.69). **(E)** Modulation frequency of the sinusoidal eye velocity response was similar between groups (*p* = 0.89). Mann-Whitney test, *N* = 4 mice per condition.

To further assess vestibulomotor function, we next tested whether otolith-mediated vestibulo-ocular reflex (VOR) behaviors were impaired in *Atoh1-Cre* conditional mutant mice, as previously observed in constitutive *Gpr156* knockouts^11^. During the off-vertical axis rotation (OVAR) task, mice were tilted 17° from vertical and rotated at a constant velocity (50°/s for 72 s). This stimulus evokes an initial canal-mediated eye velocity response that decays exponentially over ~ 10–15 s, followed by a steady-state sinusoidal response driven by the otolith organs (Fig. 8A). We found that *Atoh1-Cre* conditional mutants exhibited a significant reduction in the amplitude of the sinusoidal component of the eye movement response (Fig. 8B), consistent with a weaker otolith-driven compensatory eye movement. In contrast, the initial exponential amplitude (Fig. 8C) and time constant (Fig. 8D) did not differ significantly between groups. While mutants exhibited slightly reduced values and variability was greater (*n* = 4), these results suggest that canal-mediated velocity storage is largely preserved, although we cannot exclude subtle deficits given the limited sample size. The frequency of the sinusoidal component was comparable between mutants and controls (Fig. 8E), as expected given that the velocity of rotation was matched across groups—this frequency reflects the periodic repositioning of the head relative to gravity. Together, these findings indicate that GPR156 function is specifically required in hair cells for normal otolith-mediated VOR performance.

## Discussion

In this study, we restricted *Gpr156* inactivation to hair cells and a subset of hair cells precursors that can also generate supporting cells using a new *Gpr156* conditional allele and an *Atoh1-Cre* driver^19,20^. We demonstrated that this targeted approach recapitulates both the hair cell orientation defects and the sensory deficits previously reported in constitutive *Gpr156* mutants^3,11^. The incomplete penetrance of the misorientation phenotype in hair cell-targeted mutants plausibly explains why physiological assessments revealed similar auditory and vestibular organ impairments as in constitutive mutants, albeit with milder severity. These findings indicate that the absence of GPR156 in hair cells alone could account in large part for both hearing loss as well as deficits in swimming behavior and vestibulo-ocular reflexes observed in null mutants. Moreover, as we previously showed that GPR156-deficient hair cells retain apparently normal mechanotransduction when stimulated along their intrinsic directionality^3,11^, our results suggest that hair cell misorientation is the primary cause of the observed functional impairments. However, we cannot entirely rule out the possibility that loss of GPR156 in other cell type(s) may exacerbate physiological defects resulting from hair cell misorientation. ABR deficits are particularly severe at high frequencies (32, 40 kHz) in both *Atoh1-Cre* conditional and constitutive inactivation (Fig. 6A-B). As OHC1-2 misorientation is consistent along the cochlear duct^3^, we speculate that this defect more severely impacts the cochlear amplifier at the cochlear base (higher frequencies) due to well-described higher stiffness and dampened vibrations in this region^22^.

The transgenic *Atoh1-Cre* strain used in this study^19^ is known to leave a few sporadic hair cells unrecombined with different flox alleles^10,23^. This can account for the normally oriented auditory escaper cells that retain GPR156 protein at the medial junction (Fig. 4A-B; red arrowhead). Additionally, the presence of escapers lacking detectable GPR156 at the time of analysis (P4) can be explained by the timing of *Gpr156* inactivation. Specifically, if recombination or GPR156 depletion occurs after the critical window during which OHC1-2 break central symmetry and adopt a lateral orientation under the influence of GPR156 (E17.5 at the cochlear mid-apex^3^), some cells could maintain a lateral orientation despite eventually losing GPR156.

*Emx2* expression is required to activate GPR156 signaling^3^. Notably, constitutive *Emx2* inactivation abrogates OHC fate, preventing analysis of OHC orientation^8^. A targeted inactivation of *Emx2* in hair cells has been studied in the vestibular system, but not in the cochlea^7,17^. Based on results in this study, we expect that a specific inactivation of *Emx2* in auditory hair cells would not occur in time to silence GPR156 activity, leading to largely normal hair cell orientation. Here, by targeting GPR156, a downstream EMX2 effector, we achieved inactivation that overlaps with the period when hair cells break symmetry and adopt their normal orientation, thereby enabling a misorientation phenotype with partial penetrance.

A recent study proposed that loss of GPR156 in adult hair cells leads to their degeneration and subsequent deafness, suggesting a maintenance role for GPR156 in auditory function^24^. These conclusions were based on viral delivery of shRNAs aimed at silencing *Gpr156* expression, although the shRNAs only partially suppressed *Gpr156* in cell culture assays. It remains unclear why virally transduced hair cells would degenerate, especially given that no hair cell loss was observed in constitutive *Gpr156* mutants at young adult stages^3^. Moreover, the proposed GPR156 maintenance mechanism is difficult to reconcile with the observation that mature hair cells transduced with the shRNAs appear normally oriented, whereas constitutive mutants retain their misorientation pattern as adults^3^. The *Gpr156*^*2HA − Flox*^ strain developed in this study provides a valuable tool for conditional inactivation experiments, and will enable precise, genetically-based *Gpr156* inactivation in mature hair cells and other cell types.

## Methods

### Mouse strains and husbandry

The *Gpr156*^*del*^ strain (*C56BL/6N-Gpr156*^*tm1.1(KOMP)Vlcg/J*^; MGI:5608696) was obtained from the Knockout Mouse Project (KOMP) and studied previously in^3^. To generate the *Gpr156*^*2HA − flox*^ strain in the C57BL/6J background, we cloned a plasmid-based donor vector to add two HA tags at the *Gpr156* start codon (N-terminal) and flank exons 2 and 3 with *loxP* sites. This construct was assembled with the Gibson method and consisted in a synthesized *loxP-2HA-exon2-3 genomic region-loxP* fragment flanked by two ~ 2 kb PCR-based homology arms. The coding region was modified by introducing silent mutations to limit recombination between the *loxP* positions. The following guide RNAs were used: upstream: AACAACCTTTCACTTCTACT downstream: ACTGCAAAGCTAGTGACCAT. *Streptococcus pyogenes* Cas9 (SpCas9) V3 protein and gRNAs were purchased as part of the Alt-R CRISPR-Cas9 system using the crRNA: tracrRNA duplex format as the gRNA species (IDT, USA). Alt-R CRISPR-Cas9 crRNAs (#1072532) were synthesized using the gRNA sequences and hybridized with the Alt-R tracrRNA (#1072534) as per manufacturer’s instructions. Guide RNAs and CRISPR-Cas9 reagents were delivered in mouse zygotes via microinjection. To prepare the gene editing reagent for microinjection, SpCas9:gRNA Ribonucleoprotein (RNP) complexes were formed by incubating AltR-SpCas9 V3 (#1081059) and gRNA duplexes for 20 minutes at room temperature in embryo-tested TE buffer (pH 7.5). The SpCas9 protein and gRNA duplex were at 833 ng/µl and 389 ng/µl, respectively, during complex formation. Post RNP formation, the donor plasmid was added, and the mixture spun at 14K RPM in a microcentrifuge. The supernatant was transferred to a clean tube and stored on ice until use in the embryo microinjection procedure. The final concentration of the gRNA, SpCas9 and donor components in the microinjection mixture was 50ng/µl, 100 ng/µl and 10 ng/µl, respectively. Founder animals were tested for donor integration using PCR amplification with genomic primers outside of the homology arms. 5’ product: 5’ext_F ATTTGGCACATACTGGGCAC and HA_R GAACATCGTATGGGTATCCAGC. 3’ product: modif_ex3_F TTTCCTCGCCTTTACCATCC and 3’ext_R TTGGTTGTTATCTGTGGGCC. In order to segregate away potential non-specific mutations, positive founders were bred for two generations with C57BL/6J animals to generate a N2 heterozygote stock. The established stock was then genotyped using a 3-primer PCR strategy (Gpr156_F AACCTGCGTGTGCATGTTTG, HA_R GAACATCGTATGGGTATCCAGC, Gpr156_R TCTACCACTACCACCATCAC; *wild-type* product 575 bp, *2HA-flox* product 486 bp). The two *Cre* strains used in this work are *Atoh1-Cre* (*Tg(Atoh1-cre)1Bfri;* MGI: 3775845)^19^ and *Foxg1-Cre* (*Foxg1*^*tm1(cre)Skm*^; MGI: 1932522)^18^ and were obtained at The Jackson Laboratory. Animals were maintained under standard housing conditions (14 h light / 10 h dark cycle, ambient temperature and normal humidity). This study was conducted in accordance with ARRIVE guidelines. All animal procedures were reviewed for compliance and approved by the Animal Care and Use Committee of The Jackson Laboratory and the Johns Hopkins University School of Medicine. These include euthanasia methods: decapitation for neonates (up to P8), cervical dislocation (from P17, >~7.5 g body weight) or CO2 inhalation (from P9) for adults. No anesthesia was performed prior to euthanasia. These organizations meet the voluntary accreditation and assessment guidelines of the American Association for Accreditation of Laboratory Animal Care International, AAALAC, a private, nonprofit organization that promotes the humane treatment of animals in science.

### Immunofluorescence and antibodies

The inner ears of postnatal animals were extracted and immediately fixed in paraformaldehyde (PFA 4%; Electron Microscopy Sciences; 15710) for 1 h at 4 °C. After fixation, inner ears were dissected to isolate the cochlea and the vestibular utricle before exposing the sensory epithelium of both organs. Samples were then permeabilized and blocked in PBS with 0.5% Triton-X100 and bovine serum albumin (1%) for at least at 1 h at room temperature. Primary and secondary antibodies were incubated overnight at 4 °C in PBS with 0.025% sodium azide. Fluorescent dye-conjugated phalloidin was added to secondary antibodies. Samples were washed 3 times in PBS + 0.05% Triton-X100 after each antibody incubation, and post-fixed in PFA 4% for at least 1 h at room temperature. Samples were then mounted flat on microscopy slides using Mowiol as mounting medium (Calbiochem/MilliporeSigma 4759041), directly under a 18 × 18 mm #1.5 coverglass (VWR 48366-045). Mowiol (10% w/v) was prepared in 25% (w/v) glycerol and 0.1 M Tris-Cl pH8.5.

Primary antibodies used were:

Rabbit anti-HA (Cell Signaling Tech; 3724 S).

Rat anti-ZO1 (Developmental Studies Hybridoma Bank, R26.4 C).

Rabbit anti-Pericentrin/PCNT (Biolegend/Covance, PRB-432 C).

Mouse anti- βII-spectrin (SPNB2) (BD Transduction, 612563).

Mouse anti-acetylated alpha tubulin (Santa Cruz Biotechnolog, y scbt-23950).

Secondary antibodies from ThermoFisher Scientific were raised in donkey and conjugated to Alexa Fluor (AF) 488, 555, or 647 (donkey anti-rat AF488 (A-21208), donkey anti-rabbit AF555 (A-31572), donkey anti-mouse AF647 (A-31571)). Fluorescent conjugated phalloidins used to label F-actin were from ThermoFisher Scientific (AF488, A12379) and Biotium (CF405, 89138-126).

### Microscopy: sample cohorts, image acquisition and analysis

All quantifications include at least three animals per genotype, except for the controls in Fig. 4B (2 animals). The animal cohort size (N) and the number of hair cells (n) analyzed are indicated in all graphs or their legends. When an experimental outcome was not quantified, at least 3 mutants and control littermates across two or more litters were analyzed. Figure panels illustrate the representative outcome observed in all samples of similar genotypes.

Confocal images were captured with a LSM800 line scanning confocal microscope, a 63x NA1.4 oil objective, the Airyscan detector in confocal mode and the Zen 2.3 or Zen 2.6 software (Carl Zeiss AG). To quantify hair cell orientation (Figs. 2B, 3B, 4B and 5B), images were captured with a Leica DM5500B widefield microscope, a 63x oil objective, a Hamamatsu ORCA-Flash4.0 sCMOS camera and the Leica Application Suite (LasX) software (Leica Microsystems). All confocal images in the same experiment were acquired using the same laser intensity and gain and were then processed in Adobe Photoshop (CC2024) where the same image treatment was applied across conditions.

To determine cell orientation in the cochlea (Figs. 2B and 4B), the angle separating the longitudinal axis of the organ of Corti from a vector running from the center of mass of the hair cell to the fonticulus/base of the kinocilium was measured using the angle tool in Fiji. The fonticulus indicates the position of the basal body and was visualized as the region devoid of F-actin signals in the cuticular plate, and the kinocilium was labeled with acetylated tubulin. The acetylated tubulin channel was not displayed in figures for simplicity. In both right and left cochleae, angles were measured at the mid-cochlear position (~ 50%) so that 0° pointed towards the cochlear base and 90° towards the cochlear periphery (lateral). In the utricle lateral extrastriolar region (Figs. 3 and 5), a rectangular 120 × 60 μm ROI was drawn from the lateral edge of the macula and every hair cell in the ROI was analyzed. Cell orientation was determined by measuring the angle separating the axis of the lateral edge of the macula and a vector running from the center of mass of the hair cell to the basal body stained with PCNT. Measurements were taken so that 90° pointed towards the lateral edge, and 270° pointed towards the line of polarity reversal. The mosaic distribution of hair cells and supporting cells in Figs. 3A and 5A was established using βII-spectrin and F-actin labeling (not shown).

### Auditory tests: ABR and DPOAE

All auditory tests were performed in a sound-attenuating chamber where a heating pad (FHC Inc.) maintained the body temperature of the anesthetized animals at 37 °C. All animals were anesthetized with a mix of ketamine and xylazine (1 mg and 0.8 mg per 10 g of body weight, respectively). For Auditory Brainstem Response (ABR), mice were tested using the RZ6 Multi-I/O Processor System coupled to the RA4PA 4-channel Medusa Amplifier (Tucker-Davis Technologies) and SigGen/BioSig software (Tucker-Davis Technologies). ABR were recorded after binaural stimulation in an open field using tone bursts at 8, 16, 32, and 40 kHz and at 21 stimuli/second and a speaker located 5 cm away from the animal’s ears. A waveform was then produced by averaging the response from 512 stimuli for each frequency/dB level. Subdermal needles were used as electrodes, the active electrode inserted at the cranial vertex, the reference electrode under the left ear and the ground electrode at the right thigh. ABR thresholds were obtained by reducing the sound pressure (SPL) by 5 dB steps from 90 to 20 dB to identify the lowest level at which an ABR waveform could be detected. A value of 100dB is attributed to animals that lack a response at 90 dB stimulation (profound deafness) (Fig. 6A-B). Waveforms were compared by simultaneously displaying 5 or more dB levels at the same time on the screen. To characterize wave I, we extracted trace data measurements from BioSig, and used an R script leveraging the tidyverse, Rmisc and ggpubr packages to annotate peaks and troughs on the ABR waveform, calculating the amplitude and measuring the latency of the peak.

For Distortion Product Otoacoustic Emissions (DPOAEs), mice were tested using the RZ6 Multi-I/O Processor and SigGen/BioSig software (Tucker-Davis Technologies) to generate and control the stimuli. Pure tone frequencies (f2/f1 ratio = 1.2) at 8, 12, 16, 24 and 32 kHz and at equal levels of sound pressure (L1 = L2) were generated by the RZ6 processor and attenuated through PA5 programmable attenuators. Separate drivers were used to route these attenuated signals to mix acoustically in the ear canal with the help of an earpiece. For each animal, sound pressures were tested in 512 readings from 80 to 20 dB in 10 dB decrements. A value of 90dB is attributed to animals that lack a response at 80 dB stimulation (Fig. 6E-F). SPLs originating from the ear canal were recorded with a low-noise prone microphone (ER-10B + Microphone, Etymotic Research). The signal from the microphone was amplified 10 times and re-routed to the RZ6 processor. This acoustic signal was sampled at 100 kHz and Fast Fourier Transformations (FFTs) of the signal were averaged. This FFT waveform was utilized to measure the amplitudes of f1, f2, and the (2f1-f2) distortion product. Threshold for amplification was determined by comparing the distortion product to background levels: if the distortion product peak was higher in magnitude than any peak present in the background (noise floor), the acquired distortion product was recognized as a real signal. Readings were taken from 0 to 97.7 kHz and the noise floor was calculated by averaging all readings across this frequency window. An R script using the tidyverse package was used to determine the dB value of the distortion product peaks and to calculate the noise floor. ABR and DPOAE datasets for the constitutive mutant (*Gpr156*^*del*^) were published previously in Kindt et al. 2021^3^ and are shown for comparison only.

### Angle and auditory test plotting and statistical analysis

The circular diagrams of hair cell orientation (Figs. 2B, 3B, 4B and 5B) were generated using R (4.2.2) and Rstudio (2022.12.2 + 353). The script used can be found in Jarysta et al.^10^ and on Github at: https://github.com/Tarchini-Lab/R-code-for-circular-diagrams. The circular mean is indicated by the angle of the red line, and the length of the red arc indicates the mean circular deviation, both obtained using the colsats function of the R circular package. ABR and DPOAE thresholds were plotted in GraphPad Prism 9 and individual graphs show the mean value with standard deviation. ABR waveform (Fig. 6C), wave I amplitude and latency (Fig. 6D), and distortion products (Fig. 6G) were plotted in R (4.2.2) and Rstudio (2022.12.2 + 353) using the ggpubr package, and graphs show the mean value with standard error of the mean. The scripts used can be found on Github at: https://github.com/Tarchini-Lab/R-codes-ABR-and-DPOAE-Analysis. Data distribution between genotypes was tested for significance using 2-way ANOVA with Sidak’s multiple comparison post-hoc test for thresholds (Fig. 6A, B, E, F) and wave I amplitude and latency (Fig. 6C, D). For the distortion products (Fig. 6G), we used three-way ANOVA with a Tukey multiple comparison post-hoc test. p-values were summarized as follows: **** *p* < 0.0001, *** *p* < 0.001, ** *p* < 0.01, * *p* < 0.05, ns *p* > 0.05 (not significant).

### Swim assessments

To assess swim performance in mouse mutants and controls, we employed both standard observational scoring and objective quantification using an instrumented swim assay. Scored swim assessments were performed in a cylindrical tank approximately 8 inches wide by 10 inches tall. Mice swam for 1 min and performance was scored as follows: 0 indicated completion of swim trial without intervention, 1 indicated intermittent periods of low or no activity in the animal, and 2 indicated failure to complete the trial. Statistical comparisons were performed using GraphPad Prism 10.

For instrumented swim using an inertial measurement unit (IMU), an IMU was attached to the mouse’s head as previously described^12,25^. Mice swam in a large tank approximately 21 × 16 × 12 in (L x W x H) for three 1-minute trials with a 1-minute inter-trial interval. IMU data were sampled at 500 Hz and up-sampled to 1 kHz prior to analysis. Power spectral densities (PSDs) were computed for each axis using Welch’s method (MATLAB pwelch function; MathWorks) with a 4,096-point Hamming window (equivalent to 4.096 s) and nfft set to 4096. PSDs were calculated across all six movement dimensions. To assess statistical significance, a non-parametric permutation test was applied using a moving-frequency window approach. At each frequency bin within the 0.1–30 Hz range, PSD values were averaged within the moving window, and significance was assessed using 1,000 permutations. This analysis was conducted independently for each sensor axis. Significant differences were visualized using color-coded shading with thresholds of *p* < 0.05 (*), 0.01 (**), 0.001 (***), and 0.0001 (****). Spherical representations of head position during a trial were plotted as described in Hughes et al.^12^. Analysis of instrumented swim was performed using Matlab (MathWorks).

### Off-Vertical axis rotation

Eye movement measurements during OVAR in alert mice were performed as previously described^26^. Briefly, mice were head-fixed on a rotating platform tilted 17° relative to the ground. The platform’s speed was ramped from 0 to 50°/s over 500 milliseconds and then maintained at constant velocity for 72 s (corresponding to 10 full rotations) before stopping. Eye movements were recorded using an infrared video system (ETL-200, ISCAN). Quick phases were identified as previously described^26^ and excluded from further analysis. We then used a linear regression approach^27^ to estimate the time constant of the slow-phase eye velocity decay during OVAR, as well as amplitude and frequency of its sinusoidal modulation. Statistical comparisons were performed using GraphPad Prism 10.

## Data Availability

The research data that support the findings in this study, including detailed cohort sizes, graphed values and statistical analysis, is available in Zenodo with the identifier DOI: 10.5281/zenodo.18106827. The R codes to produce circular diagrams representing hair cell orientation are available in GitHub at https://github.com/Tarchini-Lab/R-code-for-circular-diagrams. The R codes to graph ABR wave I amplitude, latency and DPOAEs are available in GitHub at https://github.com/Tarchini-Lab/R-codes-ABR-and-DPOAE-Analysis. Instrumented swim codes are available in GitHub at https://github.com/CullenLab/SWIMU_SwimTest/tree/main.
